# Distributed source analysis of magnetoencephalography using a volume head model combined with statistical methods improves focus diagnosis in epilepsy surgery

**DOI:** 10.1038/s41598-020-62098-5

**Published:** 2020-03-24

**Authors:** Tomotaka Ishizaki, Satoshi Maesawa, Daisuke Nakatsubo, Hiroyuki Yamamoto, Sou Takai, Masashi Shibata, Sachiko Kato, Jun Natsume, Minoru Hoshiyama, Toshihiko Wakabayashi

**Affiliations:** 1 Department of Neurosurgery, Nagoya University Graduate School of Medicine, Nagoya, Aichi Japan; 2 Brain and Mind Research Center, Nagoya University, Nagoya, Aichi Japan; 3 Department of Pediatrics, Nagoya University Graduate School of Medicine, Nagoya, Aichi Japan

**Keywords:** Epilepsy, Epilepsy

## Abstract

Deep-seated epileptic focus estimation using magnetoencephalography is challenging because of its low signal-to-noise ratio and the ambiguity of current sources estimated by interictal epileptiform discharge (IED). We developed a distributed source (DS) analysis method using a volume head model as the source space of the forward model and standardized low-resolution brain electromagnetic tomography combined with statistical methods (permutation tests between IEDs and baselines and false discovery rate between voxels to reduce variation). We aimed to evaluate the efficacy of the combined DS (cDS) analysis in surgical cases. In total, 19 surgical cases with adult and pediatric focal epilepsy were evaluated. Both cDS and equivalent current dipole (ECD) analyses were performed in all cases. The concordance rates of the two methods with surgically identified epileptic foci were calculated and compared with surgical outcomes. Concordance rates from the cDS analysis were significantly higher than those from the ECD analysis (68.4% vs. 26.3%), especially in cases with deep-seated lesions, such as in the interhemispheric, fronto-temporal base, and mesial temporal structures (81.8% vs. 9.1%). Furthermore, the concordance rate correlated well with surgical outcomes. In conclusion, cDS analysis has better diagnostic performance in focal epilepsy, especially with deep-seated epileptic focus, and potentially leads to good surgical outcomes.

## Introduction

Magnetoencephalography (MEG) is a powerful tool to estimate the current source of epileptic discharge^1–3^. The irritative zone (IZ) localized by the current source of the interictal epileptiform discharge (IED) analyzed by MEG was correlated well with the IZ defined by intracranial electroencephalogram (EEG)^4^. In addition, the IZ localized by MEG showed a higher concordance rate with IZ defined by intracranial EEG than conventional EEG or high-density EEG^5^. The resection percentage of the IZ localized by MEG predicts the patient’s outcome after surgery^5^. Therefore, MEG analysis for current source of IED could be a promising non-invasive focus diagnosis alternative to intracranial EEG. The epileptogenic zone, which should be surgically removed for epilepsy control, is often located in the deep structures of the brain, such as the basal and medial temporal regions. Some epileptic lesions, such as tumors, malformations, or morphologically abnormal structures, are located in the subcortical regions rather than in the cortex. Magnetic field strength decreases proportionally to the distance between the current source and detecting coil; therefore, estimating the source localization in the deep structures of the brain is difficult^3,6,7^. A long distance between the current source and detecting coil does not mean inability to estimate the source localization, but estimation will have low reliability due to the low signal-to-noise ratio (SNR)^8^. This leads to difficulties in estimating the current source for focus detection.

A conventional method of source estimation based on the inverse model is the equivalent current dipole (ECD) analysis, which is used as the only validated and approved method in clinical practice to estimate epileptogenic generators^1–3^. ECD analysis assumes that a human head is a three-dimensional sphere where all points generate a magnetic field equally, and the current source that generates the magnetic field recorded by MEG is mathematically estimated as a current vector in one place of the spherical head space. As such, single ECD analysis is a convenient and useful method for focus diagnosis of cortical surface lesion with simple anatomical structure. However, focus diagnosis of deep-seated lesion with ECD is difficult because SNR decreases with depth, IED is widespread, sometimes through abnormal neural networks, and current source is extended^9,10^. Additionally, the ECD selection criteria vary among laboratories, and the method used is not objective^9^. To minimize the limitations of ECD analysis, advances in computing techniques resulted in the development of distributed source (DS) analysis^11^. DS as another method to estimate current sources considers anatomical brain structures and assumes that there are multiple current sources that generate current distribution in the brain space^9^. Representative methods of DS analysis have been described and include minimum norm estimation (MNE)^11^, minimum current estimate^12^, and beamformer^13^. Furthermore, the MNE-based method, L2-minimum norm^14^, dSPM^15^, and standardized low-resolution brain electromagnetic tomography (sLORETA)^16^ are used in epileptic focus diagnosis.

However, the results of the focus diagnosis by DS analysis of each IED tend to be ambiguous because the current distribution of IED estimated by the DS analysis have a certain extent, and the maximum intensity voxels of each IED are located in various areas. Thus, despite the weaknesses of ECD analysis, MEG experts prefer and continue to use ECD analysis for clinical MEG focus diagnosis. To overcome the limitations of DS analysis, we tested the forward and inverse models to assess their applicability and informative capability of identifying epileptic lesions for surgical treatment in the following steps. First, we improved the current source estimation for epileptic focus diagnosis and made it objective by combining DS analysis with statistical methods based on the difference in current distribution between IED and baseline (BL) activity and between voxels. This new method is referred to as the “combined DS (cDS) analysis” in this paper. Second, we used and improved the volume head model as source space of the forward model and sLORETA^16^ of the DS analysis as the inverse model. This study aimed to evaluate the efficacy of the combined DS (cDS) analysis in surgical cases.

## Results

### Clinical profiles

Of the 19 patients included in the final analysis, 11 patients had deep-seated lesions. Table 1 shows the clinical profiles, and Table 2 shows the results of the focus diagnosis and postoperative course. In total, 13, four, and two patients had Engel class I, class II, and III outcomes, respectively; no Engel class IV patient was reported. All cases were followed up for more than 14 months from surgery (mean, 31.8; range, 14–56).

**Table 1:** Clinical profiles.

Case	Sex / age	Epilepsy type	Seizure type and semiology	MRI lesion	Surgery	Resection	Deep-seated lesion	Pathology
1	F/19	L-FLE	LOC	+	Les	L-CgG	+IH	PA
2	F/27	L-MTLE	Olfactory aura, R-hand dystonic posture, LOC	+	SAH	L-Hip, Amy, PHG, Un	+MTL	HS
3	M/32	L-TLE	Oral automatism, head version, LOC	+	ATL	L-Hip, Amy, PHG, FuG, Un, TP, aSTG, aMTG, aITG	−	NSC
4	M/26	L-PLE	Facial abnormal sensation, LOC, sGTCS	+	Les	L-ScG	+BS	CM
5	M/62	L-NTLE	R-arm tonic seizure, LOC	+	ATL	L-Hip, Amy, PHG, FuG, Un, TP, aSTG, aMTG, aITG	+TB	HS
6	M/12	L-MTLE	Epigastric discomfort, LOC, hypermotor, head and trunk version, sGTCS	+	SAH	L-Hip, Amy, PHG, Un	+MTL	FCD type I
7	M/21	R-NTLE, OLE	Motion arrest, oral automatism, LOC, sGTCS	−	Les	R-pSTG, pMTG, pITG, IOG	−	Gliosis
8	F/21	R-FLE	sGTCS	+	Les	R-SFG, MFG	−	DNT
9	F/11	R-NTLE	Motion arrest, facial stiffness, hand automatism, LOC	−	ATL	L-Hip, Amy, PHG, FuG, Un, TP, aSTG, aMTG, aITG	+TB	FCD type I
10	F/44	R-MTLE	LOC, tonic posture	+	SAH	R-Hip, Amy, PHG, Un	+MTL	FCD type II
11	F/16	R-TLE	Motion arrest, oral automatism, L-dystonic posture, head version, LOC	+	ATL	R-Hip, Amy, PHG, FuG, Un, TP, aSTG, aMTG, aITG	−	FCD type II
12	M/15	L-TLE	sGTCS	+	Les	L-aMTG	−	GG
13	M/30	L-MTLE	LOC, sGTCS	+	SAH	L-Hip, Amy, PHG, Un	+MTL	FCD type II
14	M/44	L-MTLE	Oral and hand automatism, head and trunk version, LOC, sGTCS	+	SAH	L-Hip, Amy, PHG, Un	+MTL	HS
15	M/40	R-MTLE	L-facial spasm, head and trunk version, sGTCS	+	ATL	R-Hip, Amy, PHG, FuG, Un, TP, aSTG, aMTG, aITG	+MTL	Gliosis
16	F/20	L-TLE	Motionless staring, oral and hand automatism, LOC	+	ATL	L-Hip, Amy, PHG, FuG, Un, TP, aSTG, aMTG, aITG	−	FCD type II (temporal lobe cortex), NSC(Hip)
17	M/8	R-NTLE	Fear, vocalization, hypermotor, tonic posture	−	Les	R-aSTG, aMTG, aITG, pSTG, AnG	−	NSC
18	M/56	R-NTLE	Oral and hand automatism, LOC	+	Les	R-aSTG, aMTG, aITG	−	CM and FCD type IIIc
19	M/21	L-FLE	Hypermotor, sGTCS	−	Les	L-IFG(Or), OFG	+FB	FCD type II

Abbreviations: F, female; M, male; L-, left; R-, right; FLE, frontal lobe epilepsy; MTLE, mesial temporal lobe epilepsy; TLE, temporal lobe epilepsy; PLE, parietal lobe epilepsy; NTLE, neocortical temporal lobe epilepsy; OLE, occipital lobe epilepsy; LOC, loss of consciousness; sGTCS, secondary generalized tonic-clonic seizure; Les, lesionectomy; SAH, selective amygdalohippocampectomy; ATL, anterior temporal lobectomy; CgG, cingulate gyrus; Hip, hippocampus; Amy, amygdala; PHG, parahippocampal gyrus; FuG, fusiform gyrus; Un, uncus; TP, temporal pole; a-, anterior; p-, posterior; STG, superior temporal gyrus; MTG, middle temporal gyrus; ITG, inferior temporal gyrus; SFG, superior frontal gyrus; MFG, middle frontal gyrus; IFG (Or), orbital part of inferior frontal gyrus; OFG, orbitofrontal gyrus; AnG, angular gyrus; ScG, subcentral gyrus; IOG, inferior occipital gyrus; IH, interhemisphere; MTL, mesial temporal lobe; BS, bottom of sulcus; TB, temporal base; FB, frontal base; PA, pilocytic astrocytoma; HS, hippocampal sclerosis; NSC, no significant changes; CM, cavernous malformation; FCD, focal cortical dysplasia; DNT, dysembryoplastic neuroepithelial tumor; GG, ganglioglioma.

**Table 2:** Focus diagnosis from combined DS and ECD analysis and postoperative course.

Case	Analyzed IED number	Combined DS analysis		ECD analysis		Engel class	Follow-up (months)
		Distribution		Distribution			
1	21	L-CgG	Concordant	L-SFG	Discordant	I	59
2	9	L-Amy	Concordant	L-BG	Discordant	I	59
3	11	L-IC	Discordant	NA	Discordant	I	58
4	5	L-ScG	Concordant	L-ScG	Concordant	I	56
5	5	L-aITG	Concordant	NA	Discordant	I	47
6	3	L-Amy	Concordant	NA	Discordant	I	38
7	12	R-pITG	Concordant	R-pITG	Concordant	I	36
8	5	R-SFG	Concordant	R-MFG	Concordant	I	34
9	13	R-aITG	Concordant	NA	Discordant	I	30
10	12	R-FuG	Discordant	R-SFG	Discordant	I	25
11	8	R-aMTG	Concordant	R-FuG	Concordant	I	25
12	12	L-aMTG	Concordant	L-IFG	Discordant	I	24
13	12	L-PHG	Concordant	L-aMTG	Discordant	I	24
14	20	L-aMTG	Discordant	L-CC	Discordant	II	49
15	15	R-Hip	Concordant	NA	Discordant	II	45
16	9	L-pMTG	Discordant	NA	Discordant	II	25
17	6	R-ScG	Discordant	R-pSTG	Concordant	II	24
18	12	R-Hip	Discordant	NA	Discordant	III	28
19	16	L-OFG	Concordant	L-FP	Discordant	III	25

Abbreviations: CgG, cingulate gyrus; CC, corpus callosum; Hip, hippocampus; Amy, amygdala; PHG, parahippocampal gyrus; FuG, fusiform gyrus; a-, anterior; p-, posterior; STG, superior temporal gyrus; MTG, middle temporal gyrus; ITG, inferior temporal gyrus; FP, frontal pole; SFG, superior frontal gyrus; MFG, middle frontal gyrus; IFG, inferior frontal gyrus; OFG, orbitofrontal gyrus; ScG, subcentral gyrus; IC, insular cortex; BG, basal ganglia; NA, not available.

### Concordance rates

In all cases, the concordance rates of the cDS analysis were significantly higher than those of the ECD analysis (68.4% *vs*. 26.3%, P = 0.022). Diagnostic odds ratio was 6.07 (95% confidence interval [CI]: 1.49–24.8). The concordance rate of Engel class I cases was significantly higher in the cDS analysis than that in the ECD analysis (84.6% *vs*. 30.8%, P = 0.015). Diagnostic odds ratio was 12.4 (95% CI: 1.83–83.8). However, there were no significant differences in the concordance rates in Engel class II and III cases (cDS, 33.3% *vs*. ECD, 16.7%, P = 1.000). Diagnostic odds ratio was 2.50 (95% CI: 0.162–38.6). Sensitivity and specificity of cDS analysis to identify patients with good (Engel class I) and poor (Engel class II and III) surgical outcomes were 84.6% and 66.7%, respectively, and positive and negative predictive values of cDS analysis were 84.6% and 66.7%, respectively. On the other hand, sensitivity and specificity of ECD analysis were 30.8% and 83.3%, respectively, and positive and negative predictive values of ECD analysis were 80.0% and 35.7%, respectively. In cases with deep-seated lesions, the concordance rate of the cDS analysis (81.8%) was significantly higher than that of the ECD analysis (9.1%; P = 0.002). Diagnostic odds ratio was 45.0 (95% CI: 3.47–584). Whereas no significant difference in the concordance rates was observed in cortical surface lesion cases.

### MEG focus diagnosis and illustrative cases

**Figure 1:**
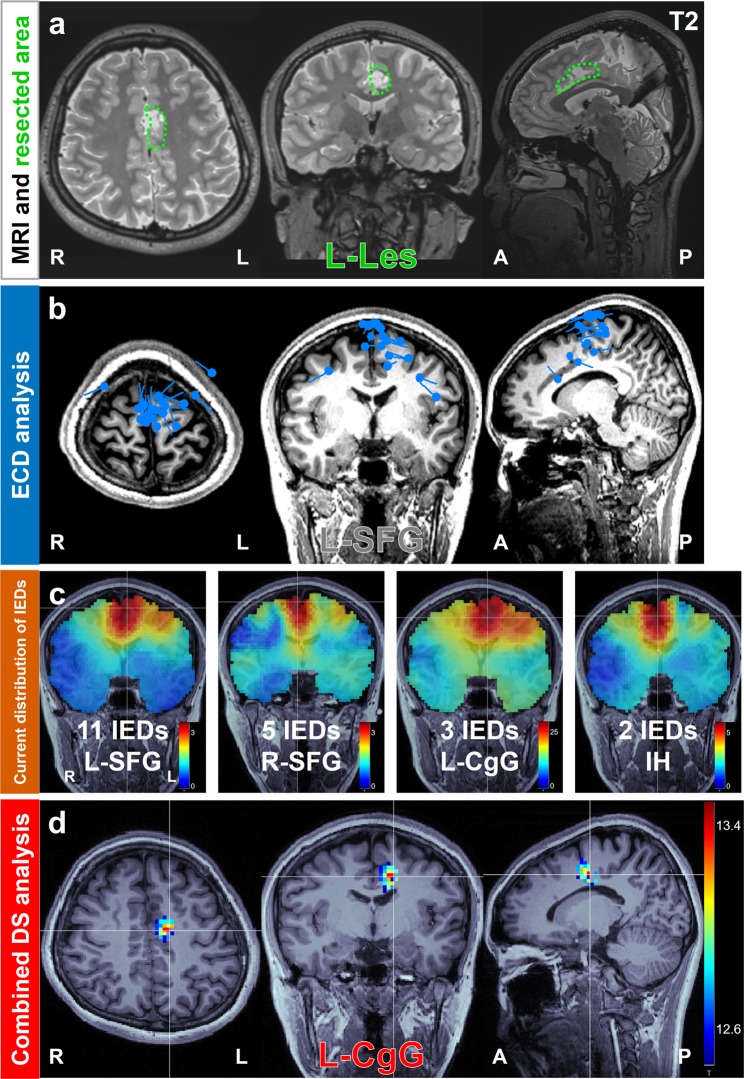
Focus diagnosis of left frontal lobe epilepsy due to pilocytic astrocytoma on the left cingulate gyrus in Case 1. (**a**) Green dotted line on the preoperative MRI represents the resected area, and the surgical method is shown. (**b**) Result of ECD analysis showing the diagnosed focus area. The estimated dipoles were localized at L-SFG, which was not concordant (gray letters) with the resected area. (**c**) Result of current distributions for 21 IEDs estimated by DS analysis using volume head model. The voxel with maximum intensity is indicated by the intersecting white lines. The current distributions were widespread, both in terms of depth and laterality. The voxels with maximum intensity were located in various areas. (**d**) Result of cDS analysis showing the diagnosed focus area. The statistically significant area was localized in L-CgG, which was concordant (colored letters) with the resected area; the seizure outcome was Engel class I. Abbreviations: ECD, equivalent current dipole; IED, interictal epileptiform discharge; DS, distributed source; cDS, combined DS; Les, lesionectomy; SFG, superior frontal gyrus; CgG, cingulate gyrus; IH, interhemisphere.

**Figure 2:**
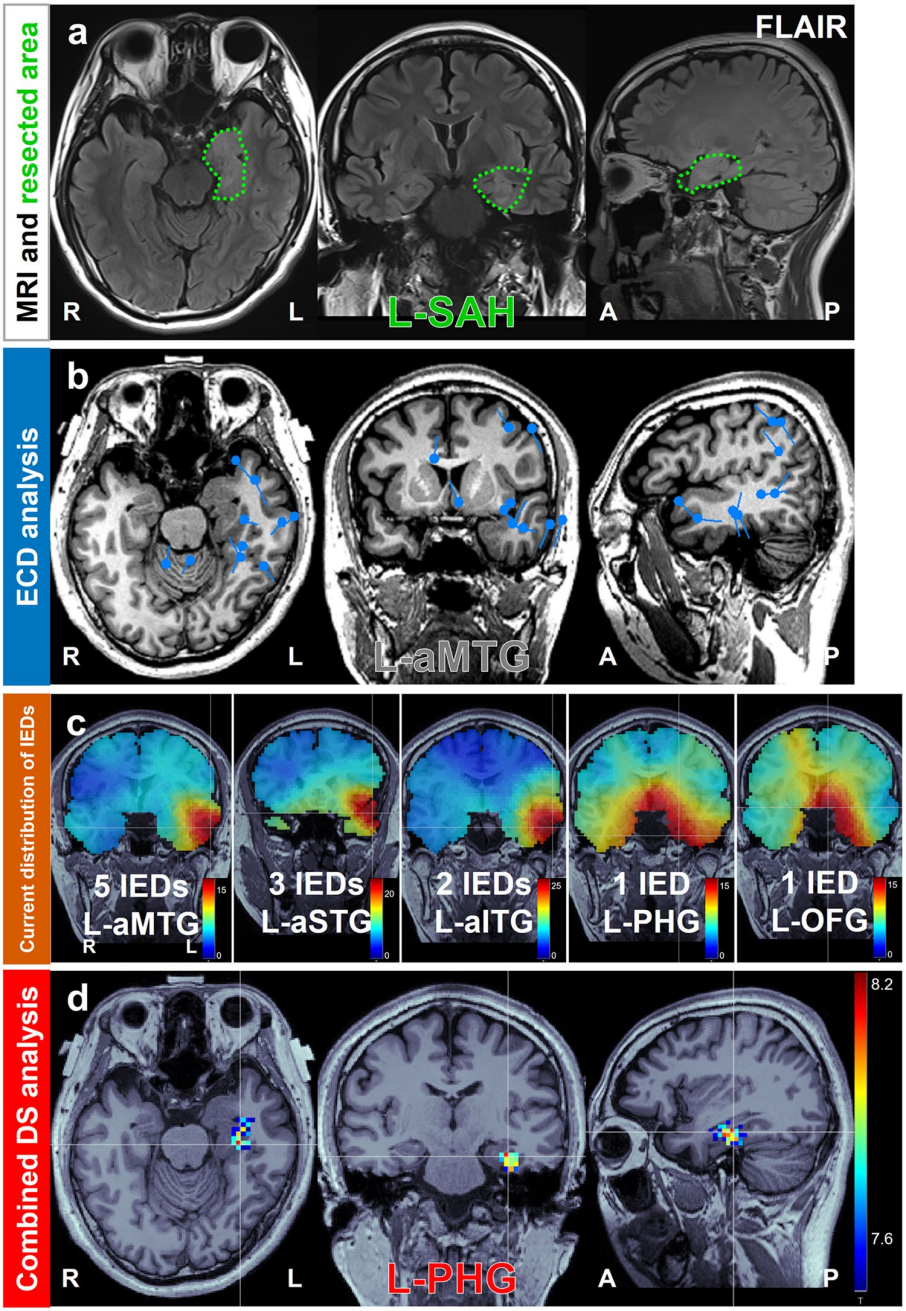
Focus diagnosis of left mesial temporal lobe epilepsy due to FCD type II in Case 13. (**a**) Green dotted line on the preoperative MRI represents the resected area and the surgical method is shown. (**b**) Result of ECD analysis showing the diagnosed focus area. The estimated dipoles were localized at L-aMTG, which was not concordant (gray letters) with the resected area. (**c**) Result of current distributions for 12 IEDs estimated via DS analysis using volume head model. The voxel with maximum intensity is indicated by a white line cross. The current distributions were widespread from the basal frontotemporal and medial temporal regions as deep area to the external temporal regions. Further, the voxels with maximum intensity were located in various areas. (**d**) Result of cDS analysis showing the diagnosed focus area. The statistically significant area was localized in L-PHG, which was concordant (colored letters) with the resected area. The seizure outcome was Engel class I. Abbreviations: FCD, focal cortical dysplasia; ECD, equivalent current dipole; IED, interictal epileptiform discharge; DS, distributed source; cDS, combined DS; SAH, selective amygdalohippocampectomy; aMTG, anterior middle temporal gyrus; PHG, parahippocampal gyrus; aSTG, anterior superior temporal gyrus; anterior inferior temporal gyrus; PHG, parahippocampal gyrus; OFG, orbitofrontal gyrus.

**Figure 3:**
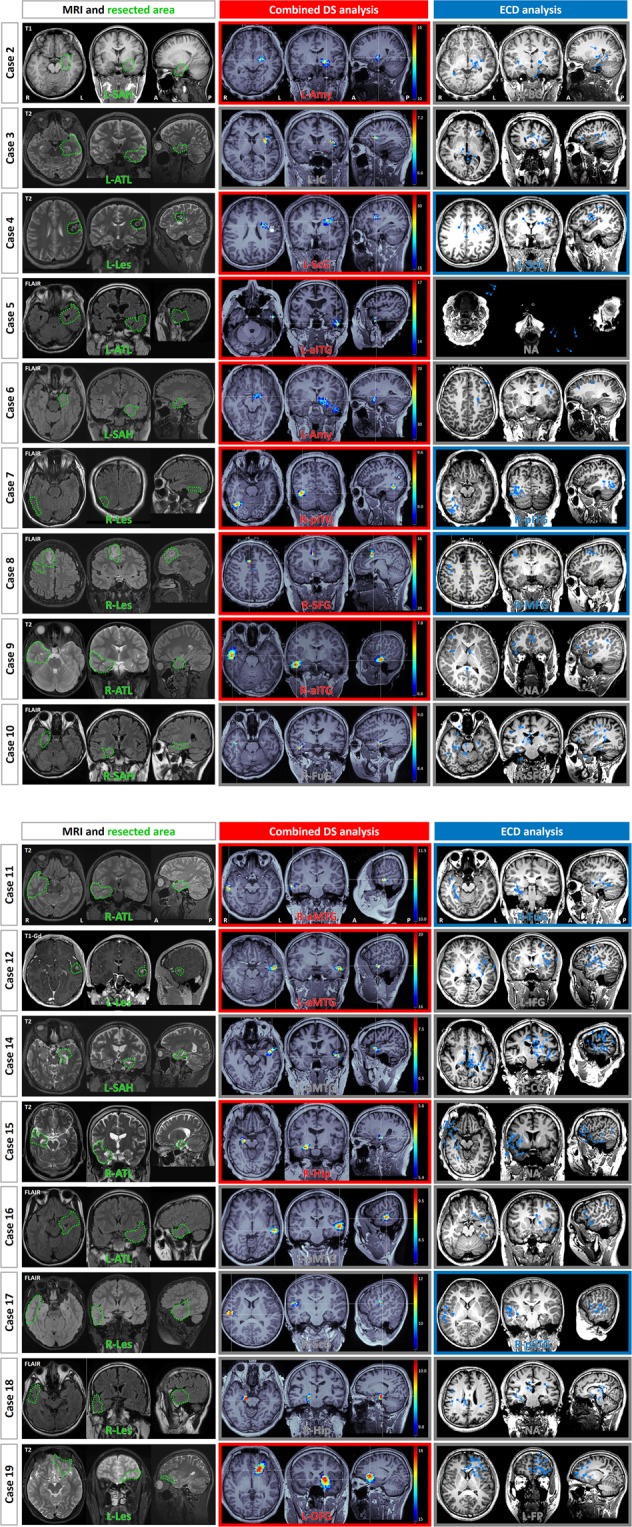
Resected area, cDS analysis, and ECD analysis of 16 cases. Left column: The green dotted line on the preoperative MRI represents the resected area and the surgical method are shown. Middle and right columns: Results of the cDS and ECD analyses showing the diagnosed focus area. When the estimated focus of cDS and ECD analyses was concordant with the resected area, the focus was described by colored letters and a colored frame. When they were discordant, the letters and frame were gray. Abbreviations: cDS, combined distributed source; ECD, equivalent current dipole; SAH, selective amygdalohippocampectomy; ATL, anterior temporal lobectomy; Les, lesionectomy; CC, corpus callosum; Hip, hippocampus; Amy, amygdala; FuG, fusiform gyrus; a-, anterior; p-, posterior; STG, superior temporal gyrus; MTG, middle temporal gyrus; ITG, inferior temporal gyrus; FP, frontal pole; SFG, superior frontal gyrus; MFG, middle frontal gyrus; IFG, inferior frontal gyrus; OFG, orbitofrontal gyrus; ScG, subcentral gyrus; IC, insular cortex; BG, basal ganglia; NA, not available.

Figures 1,2 (illustrative cases) and Fig. 3 (other cases) show the resected area from MRI scans and the results of the focus diagnosis using the cDS and ECD analyses. Focus estimation as presented in these illustrative cases is generally difficult because the foci were in deep-seated lesions. We calculated current distributions by DS analysis using the volume head model for each IED (Figs. 1c and 2c). The current distributions were well calculated in the deep area, but they spread to some extent. Variations were observed in the voxels with maximum intensity. Therefore, it was difficult to diagnose the focus as one area objectively. Then, we combined the two statistical methods with DS analysis. The cDS analysis showed that the statistically significant current distribution was localized well, and the maximum intensity voxel was in the resected area (Figs. 1d and 2d).

### Case 1

A 19-year-old woman has been having sudden motion arrest and loss of consciousness since age 15 years (Fig. 1). MRI revealed a tumor in the left cingulate gyrus. The patient was diagnosed with focal epilepsy with lesion-related impaired consciousness based on video EEG monitoring and according to positron emission tomography findings. Accordingly, we performed lesionectomy. The seizure outcome was classified as Engel class I at 1 year after surgery, and pilocytic astrocytoma was pathologically diagnosed.

In this case, conventional EEG and ECD analysis could not precisely estimate the epileptic focus because of the deep-seated interhemispheric lesion. Those conventional methods represented the focus as the cortical surface area. For example, ECD analysis indicated that the best goodness of fit (GOF) ECD cluster was scattered at the center of the cortical surface, which was not included in the resected area (Fig. 1a–b). Current distributions calculated via DS analysis using the volume head model for 21 IEDs spread from the bilateral superior frontal gyrus to bilateral cingulate gyrus, and the maximum intensity voxel varied (Fig. 1c). However, cDS analysis revealed that the statistically significant current distribution was localized adjacent to the posterior side of the tumor in the left cingulate gyrus and was consistent with the resected area (Fig. 1d). This area was resected surgically, and the patient did not develop seizures for 4 years. Thus, the area indicated by the cDS analysis was surgically proven to be the epileptic focus.

### Case 13

A 30-year-old male patient was diagnosed with focal impaired awareness seizures at age 24 years (Fig. 2). MRI revealed a tumor-like lesion in the left mesial temporal lobe. The prescribed anticonvulsants did not adequately control the seizures. After presurgical evaluation, we performed selective amygdalohippocampectomy, and the outcome was classified as Engel class I. Based on pathological examinations, we diagnosed the patient with focal cortical dysplasia (FCD) type II.

ECD analysis indicated that the left anterior middle temporal gyrus was the epileptic focus, which was discordant with the resected area (Fig. 2a,b). The current distributions and their maximum intensity voxel calculated before applying statistical methods varied widely from the deep basal fronto-temporal and medial temporal region to the external temporal region (Fig. 2c). cDS analysis revealed that the statistically significant current distribution was localized to the left parahippocampal gyrus, in agreement with the resected area (Fig. 2d). The cDS analysis precisely diagnosed the epileptic focus in the parahippocampal gyrus, i.e., an extrahippocampal area.

## Discussion

**Figure 4:**
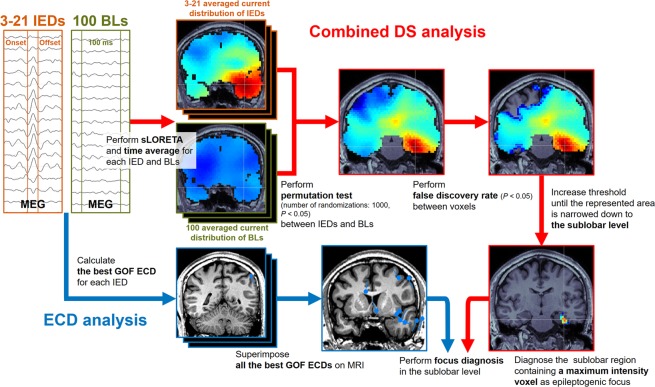
Schematic representation of the cDS and ECD analyses. IEDs were visually identified, and 100 BLs were selected from MEG recordings that underwent sLORETA followed by time averaging. Next, statistical analysis using the permutation test and false discovery rate was performed. The regions with significantly different current distributions were focused into one gyrus by increasing the statistical threshold. The sublobar region containing a maximum intensity voxel indicated by the intersecting white lines was diagnosed as epileptogenic focus. ECD analysis was performed according to the best GOF ECD cluster method. Abbreviations: cDS, combined distributed source; ECD, equivalent current dipole; IED, interictal epileptiform discharge; BL, baseline; sLORETA, standardized low-resolution brain electromagnetic tomography; GOF, goodness of fit.

In this study, we found significant differences in current distribution between IEDs and BLs (Fig. 4). Unlike previous studies^6,7,17–22^ on focus diagnosis using DS analysis, all 19 cases analyzed were consecutive surgical cases, and their number was sufficient for statistical analysis (Supplementary Table S2). We improved the DS analysis by introducing statistical methods. Previously, because of SNR reduction, current source estimation in deep brain regions was difficult^3,6,7,23^. Epileptic discharge is masked by background noise because the amplitude of the detected discharges in deep regions is attenuated. For example, Stefan *et al*. detected the epileptic discharge generated from deep regions, such as the mesial temporal lobe, by averaging the spikes and increasing the SNR^3^. Furthermore, current distribution estimated by DS analysis shows a certain extent and the point with maximum intensity of current distribution varies between IEDs. It is difficult to objectively decide on a focus area. To overcome these problems, we performed mean value difference tests of the current distributions between IEDs and BLs and multiple comparisons within each voxel with a significant current distribution (Fig. 1d, Fig. 2d, and Fig. 3). To the best of our knowledge, this is the first study to use two statistical analyses for focus diagnosis of DS analysis.

Axial gradiometers are placed on the scalp to detect magnetic fields generated by the postsynaptic discharge of pyramidal neurons located parallel to the surface of the detecting coils. Based on this principle, by constraining the dipole orientation parallel to cortical surface and modelling current distribution limited to the cortical surface, it is possible to precisely and efficiently calculate current distribution. In previous MEG studies^6,7,17–22^ that used DS analysis for focus diagnosis, a cortical surface model was used as the source space in the forward model (Supplementary Table S2). However, when examining actual surgical cases, either (i) the brain structure was deformed by lesions caused, for example, by a tumor or trauma; (ii) the tumor had an epileptogenicity (e.g., dysembryoplastic neuroepithelial tumors and gangliogliomas); (iii) the epileptic focus was in a deep area, such as the medial, basal, and subcortical regions; or (iv) the focus had a complex histological structure. Therefore, the cortical surface model could not represent the appropriate current distribution in all cases.

We hypothesized that the volume head model, which assigned the dipole with unconstrained orientation to the cortical surface and subcortical source space, could represent and evaluate the current distribution. We used the volume head model as the source space of the forward model, which was implemented in Brainstorm and is generally used in EEG/MEG analysis^24^. While the volume head model prevents problems with accuracy of cortical surface reconstruction and the lack of subcortical areas, it also means the inverse problem is less constrained, which results in lower spatial resolution. Hence, we examined the concordance between focus estimated using the volume head model and focus certified by actual surgical cases that included various anatomical structures. We found that the focus estimated via the cDS analysis using the volume head model was significantly concordant with the focus identified in actual surgical cases (Engel class I cases, 84.6%) and the deep-seated focus (81.8%). It was clinically shown that the cDS analysis using the volume head model remained accurate in the deep area. In previous studies^6,7,17–22^, brain lesions were mostly superficial, and the source space was calculated from the cortical surface model. This is the rare study to assess clinically the efficacy of the volume head model as the source space in epileptic focus diagnosis of actual surgical cases (Supplementary Table S2).

There are MNE, dSPM, and sLORETA approaches that are used for DS analysis. MNE is a typical method of DS analysis; the L2-minimum norm, the most basic method of MNE, finds the inverse matrix that minimizes the sum of squares of dipole moments^14^. However, this method tends to assign the current source toward sensors, and the accuracy of the deep structure is significantly reduced^10,25^. dSPM and sLORETA have been improved using noise normalization^10^. As such, MNE, dSPM, and sLORETA show similar current distributions. However, Hauk *et al*. showed that dSPM and sLORETA were superior to MNE in the Sylvian fissure and orbitofrontal cortex, and sLORETA was superior to dSPM in the anterior temporal lobe^26^. In focus diagnosis in epilepsy surgery, these frontal and temporal lobe regions are often included as lesions to be analyzed; hence, sLORETA was considered more suitable than MNE and dSPM. Furthermore, to investigate the inverse problem of EEG source localization, Grech *et al*. compared weighted MNE, LORETA, sLORETA, and shrinking LORETA FOCUSS using Monte-Carlo analysis. They showed that sLORETA had the fewest localization errors and ghost sources^27^. Therefore, we selected sLORETA for DS analysis as our inverse model. Conventional ECD analysis often excludes the dipole considered inappropriate for objective focus diagnosis by setting various conditions or correcting the position of the dipole located in the white matter^6,28^ and referenced magnetic field topographic map when selecting dipoles^7^. However, such subjectivity for dipole selection may cause ambiguous results. In this study, we found the best GOF ECDs for all IEDs referencing only MEG waveforms and not a magnetic field topographic map. ECDs from focus diagnosis that considered estimation errors were not excluded if the estimated dipoles were localized in the white matter or extra-axial space. Thus, the concordance rates of the ECD analysis were much lower than those of the cDS analysis because ECD analysis was performed objectively.

All cases were classified as Engel class I–III after surgical intervention. The concordance rates of the cDS analysis were higher than those of the ECD analyses in all Engel I cases and in cases with deep-seated lesions. These results indicate the efficacy of the cDS analysis. Patients with Engel class II and III outcomes did not achieve seizure-free status, and the epileptic focus, possibly, was not located in the resected areas. This could explain the lack of significant differences in concordance rates between the analytical methods.

The current source of the IED analyzed by MEG have good correlation with IZ (concordance rate 77.3%), but relatively poor correlation with seizure onset zone (concordance rate 32.8%) defined by intracranial EEG and lower sensitivity in identifying all IZs of intracranial EEG^4^. Since the cDS analysis used IED, if the result of the cDS analysis as a preoperative focus diagnosis was different from the seizure onset zone indicated by the intracranial EEG or not available, it was necessary to compare it with other modalities and consider the interpretation of the result obtained with the cDS analysis.

Our study had several limitations. We studied patients with focal epilepsy undergoing surgery; however, patient pathology varied. This population represented the clinical population undergoing epilepsy surgeries, and our data were recorded from a single center and involved a single neurosurgeon. Because the number of cases was insufficient to divide and analyze according to different pathologies, consecutive cases were chosen to ensure reliability of the clinical data. Five cases were excluded from the statistical analysis because they had only one or two IEDs. IEDs that were initially identified and collected were visually inspected by three epilepsy experts. Therefore, our method of IED selection can be better objectified by using an automatic spike detection program. In this study, the efficacy of cDS analysis was evaluated clinically using epilepsy surgery cases, however not mathematically evaluated by simulation. Simulations might evaluate the efficacy of cDS analysis from different perspectives than clinical ones. In the cDS analysis, a mean value difference tests between IEDs and baselines was performed. However, there is an existing method such as automated spike clustering and averaging, and it is desirable to be able to compare and evaluate cDS analysis with these existing methods in the future. The ECD analysis used for comparison is the method conventionally used in our institute. This ECD analysis is a general and simple; however, it is not a more sophisticated and complicated method such as multiple signal classification (MUSIC)^29^, thus the accuracy of focus diagnosis of ECD analysis may be underestimated.

## Conclusion

We identified regions with statistically significant current distribution of the IED recorded via MEG using the volume head model as the forward model and statistical methods applied to sLORETA of the DS analysis as the inverse model. The concordance rates obtained with this cDS analysis were significantly higher than those of the ECD analysis, especially in cases with deep-seated lesions, such as in the interhemispheric, fronto-temporal base, and mesial temporal structures. Furthermore, the concordance rates correlated well with surgical outcomes. Collectively, these findings indicate that cDS analysis has better diagnostic performance for deep-seated epileptic foci.

## Methods

### Patients

We retrospectively evaluated 25 consecutive patients who underwent preoperative MEG and lesionectomy, including standard temporal lobectomy and selective amygdalohippocampectomy, for adult and pediatric drug-resistant focal epilepsy at our institute between May 2014 and January 2018. Of the 25 eligible patients, 6 patients were excluded because DS analysis with statistical methods could not be performed because only one or two IEDs were identified during the recording period (n = 5) and a head model could not be constructed due to technical problems (n = 1). Finally, 19 patients (76%; 12 men; mean age at surgery 27.6 years, range, 8–62) were included in the cDS analysis.

Focus was diagnosed based on preoperative scalp EEG (10–20 system), video EEG monitoring, magnetic resonance imaging (MRI), positron emission tomography, intracranial EEG, electrocorticogram, and pathology findings. We defined cases with epileptic focus in the mesial temporal lobe, frontal and temporal base, interhemispheric, and bottom of sulcus lesions as deep-seated lesions. The seizure outcomes of these patients were evaluated using Engel’s classification^30^ at 2 years after surgery. This research was approved by the ethical committee of our institute (No. 1005-2). Informed consent was obtained from the patients and from their parents for those aged under 18 years. All methods were performed in accordance with the relevant guidelines and regulations.

### MEG

Magnetic signals at rest with closed eyes, including those in stage 1 to 2 of the sleep state, were recorded for each participant using a whole-head MEG system with 160 axial gradiometers (PQ1160C, Ricoh Co., Japan). No drug was used to induce sleep. Magnetic responses were filtered using a 1–2000 Hz initial band-pass filter and digitized with a sampling frequency of 5,000 Hz. Electroencephalography at eight scalp areas (i.e., F3, F4, C3, C4, T3, T4, P3, and P4) according to the international 10–20 system and electrocardiogram were simultaneously performed with a 1–100 Hz band-pass filter at the same sampling rate. Each participant completed 5–11 recording sessions of 4 minutes each. The MEG and EEG signals were visually observed on monitors in real time during the recording. When IEDs were detected on the monitor more than 10 times, sufficient for further analyses, the recording was completed in five sessions; otherwise, the recording was extended for 45 minutes.

### MRI and forward model estimation

MRI T1-weighted sagittal sections (slice thickness, 1.0 mm; TE, 2.5 ms; TR, 2500 ms; 192 slices) were obtained using a 3 T MR scanner (Siemens, 3 Tesla System, Erlangen, Germany). We processed the brain structure extraction from MRI using the BrainSuite open source software (Signal and Image Processing Institute, Department of Electrical Engineering Systems, University of Southern California, Los Angeles, USA)^31^. Meanwhile, the computed head model was developed based on the processed data using the Brainstorm open source software from the same institute^24^. We used the entire brain volume head model of each patient as the source space. Overlapping spheres were used for the MEG forward modelling^32^.

### Distributed source analysis and statistical analysis

A summary of the cDS and ECD analyses is shown in Fig. 4. DS and statistical analyses were performed using Brainstorm. The recorded MEG data were preprocessed using a band-pass filter of 10–50 Hz to analyze in the band where the IED spikes appear, as in previous studies^6,18,19,28^. The MEG recordings taken during sleep stage 2 were considered as the object of these analyses. The IEDs that were initially identified and collected were visually inspected by three epilepsy diagnosis and surgical treatment experts (TI, SM, and YH). IED spike onset and offset times were recorded. Reproducible spikes were chosen for analysis on unanimous agreement. On the other hand, non-reproducible spike-like activities that appear only once in the recording were excluded only when it was judged nonepileptic on unanimous agreement. Subsequently, 100 100-ms BL time windows, excluding noise, and 1-s windows pre- and post-IEDs were identified from the MEG data.

We used sLORETA^16^ as the inverse model for the time windows of IEDs and BLs. We calculated the time average of the estimated current distributions at each time window. Next, these averaged current distributions underwent a permutation test, with 1,000 randomizations, to detect significant differences in current distribution areas between IEDs and BLs, and the false discovery rate for multiple comparisons was calculated to identify significant area clusters in the current distribution between voxels. The significance level was set to *P* < 0.05. When an area determined as one of 34 sublobar regions (Supplementary Table S1) included a maximum intensity voxel, the area was diagnosed as epileptogenic focus.

### Equivalent current dipole analysis

IEDs, identified following the cDS analysis, were subjected to ECD analysis using the MEG Laboratory (Yokogawa Electric Corporation, Japan). For each IED, the ECD was computed every 5 ms from spike onset to offset. ECDs with a goodness of fit (GOF) > 70% and dipole moment Q-value between 50 nAm and 500 nAm were accepted^33^. We defined the sublobar region, including the highest number of best GOF ECDs, as the epileptic focus. When the best GOF ECDs were evenly scattered and a localized sublobar region could not be identified, we considered no epileptic focus to be available.

### Concordance rates of focus diagnosis and statistical analysis

We defined the concordance rates as the fraction of cases in which the sublobar region diagnosed as the focus by the cDS or ECD analysis was included in the actual surgical resection area defined based on postoperative MRI. The concordance rate was calculated for four subgroups: all cases, Engel class I cases, Engel class II and III cases, and cases with deep-seated lesions. Fisher’s exact test was used to compare the concordance rates of the cDS and ECD analyses. Statistical analysis was performed using IBM SPSS Statistics (International Business Machines Corporation, USA), and *P* < 0.05 was considered statistically significant.

## Supplementary information

Supplementary information.

## Data Availability

The datasets generated during and/or analysed during the current study are available from the corresponding author on reasonable request.
