# Supplementary material for: Distributed source analysis of magnetoencephalography using a volume head model combined with statistical methods improves focus diagnosis in epilepsy surgery
Source: Sci Rep. 2020 Mar 24;10:5263. doi: 10.1038/s41598-020-62098-5 (PMC7093400; doi:10.1038/s41598-020-62098-5)
Supplement: Supplementary file 1 — Supplementary information. [file 41598_2020_62098_MOESM1_ESM.pdf]

Supplementary Table S1 and S2

Distributed source analysis of magnetoencephalography using a volume head model combined with statistical methods improves focus diagnosis in epilepsy surgery

Tomotaka Ishizaki1, Satoshi Maesawa[1,2], Daisuke Nakatsubo[1,2], Hiroyuki Yamamoto[2,3], Sou Takai[1], Masashi Shibata[1], Sachiko Kato[1], Jun Natsume[2,3], Minoru Hoshiyama[2], Toshihiko Wakabayashi[1]

[1]Department of Neurosurgery, Nagoya University Graduate School of Medicine, Nagoya, Aichi, Japan
[2]Brain and Mind Research Center, Nagoya University, Nagoya, Aichi, Japan
[3]Department of Pediatrics, Nagoya University Graduate School of Medicine, Nagoya, Aichi, Japan

Supplementary Table S1: Thirty-four sublobar regions

Cerebral lobes	Sublobar regions
Frontal lobe	Superior frontal gyrus, middle frontal gyrus, inferior frontal gyrus (orbital, triangular, opercular part), precentral gyrus, frontal pole, orbitofrontal gyrus, cingulate gyrus, corpus callosum
Temporal lobe	Anterior- (dominant side: temporal pole ~35 mm; non-dominant side: ~45 mm), posterior-, superior temporal gyrus; middle temporal gyrus; inferior temporal gyrus; temporal pole; uncus; hippocampus; amygdala; parahippocampal gyrus; fusiform gyrus
Parietal lobe	Postcentral gyrus, subcentral gyrus, superior parietal lobule, supramarginal gyrus, angular gyrus, precuneus
Occipital lobe	Superior occipital gyrus, inferior occipital gyrus, cuneus, lingual gyrus
Other regions	Insular cortex, basal ganglia

Supplementary Table S2: Summary of the recent representative reports on DS analysis

Ref. No.	Year	Journal	Author	DS analysis (inverse model)	Head model (forward model)	Interictal / ictal	Surgical cases / total cases	Patients and pathology	Deep-seated lesion	Methods	Results
This paper	2020	*Scientific Reports*	Ishizaki et al.	sLORETA	Volume head model	Interictal	25/25	Patients with intractable epilepsy who underwent preoperative MEG and resections; included 11 deep-seated lesions	+	sLORETA was compared with the volume head model using statistical and ECD analyses. Concordance rate was evaluated for seizure outcome and deep-seated lesions.	Concordance rate of the modified DS analysis was significantly higher than that of the ECD analysis at 68.4% in all cases, 84.6% in Engel class I cases, and 81.8% in deep-seated lesion cases.
21	2009	*Epilepsy Research*	Tanaka et al.	dSPM	Cortical surface model (BEM)	Ictal	2/5	Patients with frontal lobe epilepsy	-	Compared ictal dSPM, interictal dSPM, and interictal ECD analysis. Ictal epileptic discharges were selected from the early part of ictal MEG.	In all spikes obtained from the early ictal discharges, dSPM localized activity consistent with the clinically determined ictal onset zone at a lobar level, except for one spike in one patient.
18	2013	*NeuroImage Clinical*	Kanamori et al.	MNE	Cortical surface model (BEM)	Interictal	7/7	Patients whose epileptogenic zones were assumed to be located on the convexity of the cerebral cortex	-	ECD, MNE, and ECoG data were compared. First, ECD analysis was performed to assess IEDs; dipoles at inappropriate locations were excluded. Second, DS analysis was performed using MNE.	MNE detected the initial portion of epileptic activity more accurately than ECD. MNE represented a similar pattern of epileptic discharge in recorded ECoG.
20	2016	*Frontiers in Neural Circuits*	Shirozu et al.	GMFT	Cortical surface model	Interictal	12/12	Patients who underwent surgical resection using extraoperative IEEG evaluation	+	Simultaneous MEG-iEEG recording was performed. Interictal epileptic spikes were analyzed by GMFT of MEG and VT of IEEG.	Overall, 10 patients (83.3%) exhibited good concordance between GMFT and VT. GMFT represented brain activity in the lateral cortices accurately, whereas deep brain regions were not represented accurately because GMFT shows only lateral neocortex activity.
7	2017	*Clinical Neurophysiology*	Shirozu et al.	GMFT	Cortical surface model	Ictal	13/13	Patients with neocortical epilepsy who underwent cortical resections and/or multiple subpial transection	-	Applied GMFT and ECD to identify the IOZ and compared GMFT and ECD with the IOZ derived from IEEG.	In 10 patients (83.3%), the IOZ detected by GMFT was concordant with that derived from IEEG. GMFT was significantly superior to ECD with respect to the detective capability and precision of delineation of ictal MEG activity.
6	2018	*Human Brain Mapping*	Pellegrino et al.	cMEM	Cortical surface model (BEM)	Interictal	31/49	49 patients whose epileptic foci were identified via a combination of IEEG and epileptogenic MRI; 31 with cortical, surgically resected lesions	+	Compared cMEM and ECD analyses. MEG spike peaks were marked and grouped according to morphology and topography. ECD in subcortical areas was projected to the closest cortical vertex.	Sublobar concordance rate with epileptic focus of cMEM was significantly higher than that of ECD in 81% of all cases and in 84% of extratemporal cases. However, there were no significant differences in temporal cases (cMEM 77% vs. ECD 73%).
19	2018	*Clinical Neurophysiology*	Nakajima et al.	Advanced dSPM	Cortical surface model (BEM)	Interictal	12/15	Patients with FCD at the bottom of sulcus	+	Developed dSPM of Advanced dSPM (AdSPM) and compared AdSPM and ECD analyses. First, dipoles were estimated using the single moving dipole method, and dipole source locations were accepted to include whether source solutions met certain criteria. Analyzed ±50 ms epochs relative to each dipole derived from the single moving dipole method via AdSPM.	AdSPM was localized on FCD at the bottom of the sulcus in 12 patients (80%), whereas ECD cluster was localized in 6 patients (40%). Overall, 10 patients (91%) showed the AdSPM spike source included in the resection area.
17	2018	*JAMA Neurology*	Alkawadri et al.	MNE	Cortical surface model	Ictal	16/44	44 patients who experienced epileptic auras or magnetic seizures, of whom 16 underwent surgical resection of the suspected focus	ND	Compared L2-MNE and ECD analyses of ictal MEG.	Resection of the areas delineated by L2-MNE correlated with better surgical outcomes than those delineated by ECD.
22	2018	*Journal of Clinical Neurophysiology*	Tanaka et al.	MNE	Cortical surface model (BEM)	Interictal	7/8	Patients who underwent MEG and subsequent IEEG monitoring for presurgical evaluation	ND	Compared MNE and IEEG analyses. Calculated the sensitivity of MEG relative to IEEG = overlapping MEG- and IEEG-active region / IEEG-active region.	The sensitivity of MEG relative to IEEG was 0.65-0.85.

DS, distributed source; sLORETA, standardized low-resolution brain electromagnetic tomography; dSPM, dynamic statistical parametric mapping; MNE, minimum norm estimates; GMFT, gradient magnetic-field topography; cMEM, coherent maximum entropy on the mean; BEM, boundary element method; FCD, focal cortical dysplasia; ECoG, electrocorticogram; IEEG, intracranial electroencephalography; VT, voltage topography; ECD, equivalent current dipole; IOZ, ictal onset zone; ND, not described
